# The effect of computerized cognitive training and transcranial direct current stimulation on working memory among post-stroke individuals: a systematic review with meta-analysis and meta-regression

**DOI:** 10.1186/s12883-024-03813-x

**Published:** 2024-09-04

**Authors:** Csaba Kazinczi, Krisztián Kocsis, Katalin Boross, Mihály Racsmány, Péter Klivényi, László Vécsei, Anita Must

**Affiliations:** 1 Department of Neurology, University of Szeged, 6, Semmelweis Street, Szeged, 6725 Hungary; 2 Department of Clinical Psychology, Semmelweis University, 25, Üllői Street, Budapest, 1091 Hungary; 3 Department of Neurology, St. Borbala Hospital, 77, Dózsa György Street, Tatabánya, 2900 Hungary; 4 University of Szeged, Institute of Psychology, 2, Egyetem Street, Szeged, 6722 Hungary; 5 HUN-REN-SZTE Neuroscience Research Group, University of Szeged, 2, Szikra Street, Szeged, 6725 Hungary; 6 WCG Clinical Endpoint Solutions, Princeton, NJ USA; 7 Department of Psychiatry, Whanganui District Health Board, Whanganui, New Zealand; 8 Department of Radiology, University of Szeged, 2, Semmelweis Street, Szeged, 6725 Hungary; 9 Institute of Cognitive Neuroscience and Psychology, HUN-REN Research Centre for Natural Sciences, 2, Magyar Tudósok Boulevard, Budapest, 1117 Hungary; 10 Cognitive Medicine Research Group, Competence Centre for Neurocybernetics of the Life Sciences Cluster, Centre of Excellence for Interdisciplinary Research, Development and Innovation, University of Szeged, 13, Dugonics Square, Szeged, 6720 Hungary

**Keywords:** Computer-based, Cognitive training, tDCS, Stroke, Working memory, Systematic review

## Abstract

**Background:**

Working memory (WM) impairment is a common phenomenon after stroke; however, its management in rehabilitation is less researched. This systematic review and meta-analysis aimed to provide a quantitative synthesis of the impact of computerised cognitive training (CCT) and transcranial direct current stimulation (tDCS) on WM span in post-stroke individuals.

**Methods:**

The literature search in PubMed, Embase, Scopus, and Cochrane Library focused on randomized controlled trials testing the effect of CCT and tDCS on treated stroke patients as compared to untreated controls. Neuropsychological instruments such as Digit Span Forward/Backward and Visual Span Forward Tests defined the outcome of WM span. After extracting study characteristics and quality assessment using the Cochrane Risk of Bias Tool, we conducted a meta-analysis and meta-regression using standardised mean differences.

**Results:**

The search yielded 4142 articles, nine of which (*N* = 461) fulfilled the inclusion criteria. In the case of CCT, we found significant improvement in Digit Span Backward Test (Z = 2.65, *P* = 0.008; 95% CI [0.10, 0.67]) and Visual Span Forward Test performance (Z = 3.05, *P* = 0.002; 95% CI [0.15, 0.69]), while for tDCS, we could not find a sufficient number of studies for the analysis. Furthermore, no significant moderating factor was found in the meta-regression.

**Conclusions:**

In conclusion, CCT appears to be a suitable choice to enhance WM span performance after stroke. However, further research is needed to investigate the effect of tDCS due to the limited number of studies.

**Trial registration:**

The meta-analysis was conducted according to PRISMA (Preferred Reporting of Systematic Reviews and Meta-Analyses) standards with a PROSPERO registration protocol (ID: CRD42023387182).

**Supplementary Information:**

The online version contains supplementary material available at 10.1186/s12883-024-03813-x.

## Background

Cognitive dysfunction after a stroke is a common phenomenon that affects a significant proportion of patients, impacting their quality of life [1]. Cognitive dysfunction typically influences language and memory domains, frequently working memory (WM) [2]. WM is responsible for processing information ‘online’ and executing goal-directed behaviour, playing a crucial role in daily activities [3]. According to theoretical considerations, one of the subdomains related to WM is the central executive (CE), which is responsible for executive functions (EF), such as updating, shifting, and inhibition [4]. Information to be processed is temporarily stored in the phonological loop (verbal modality) and the visuospatial sketchpad (visual modality). Additionally, the episodic buffer integrates diverse information in WM and is linked to long-term memory [4–6]. According to Lugtmeijer and colleagues (2021), differentiation can be made not only in terms of modality but also of WM load. WM load can be categorised into two segments: low-load WM tasks, typically demanding short-term memory processes, and high-load WM tasks, which more specifically require demanding EFs and other cognitive functions (e.g., inhibition, attention, and interference control) [7].

Various neuropsychological tests are available to assess WM functions based on load and modality. Since post-stroke cognitive dysfunction impacts WM, it is essential to consider the types of assessments with low cognitive load while being well-suited for clinical practice. In clinical settings, WM measures are often associated with specific subtests of the Wechsler Adult Intelligence Scale (WAIS), such as span tasks [8–10], which mainly measure the capacity/span of WM [11]. A relevant approach for assessing WM span involves using the Digit Span Forward test (low-load, verbal modality) and the Visual Span Forward/Corsi Block Tapping Task (low-load, visual modality) [12]. Additionally, the Digit Span Backward test is widely used in clinical settings and is considered a high-load task requiring EFs. However, compared to other high-load WM tasks (e.g., N-back, Stroop tasks), it involves significantly less cognitive load and is not recommended to be used interchangeably with other high-load measures of WM [13]. This perspective is supported by an EEG correlates study by Scharinger and colleagues (2017), which suggests that, for instance, the high-load ‘N-back’ task places notably higher demands on WM processing and cognitive control than complex span tasks. This finding has been confirmed by other research in the last decades [14–16], showing the difference between WM span tasks and other, more complex WM measurement procedures. In our study, we define a test as having a low cognitive load if it requires minimal additional processing beyond the primary task. Although tasks like the digit span test may be challenging for patients with WM deficits, these tests are more appropriate for minimising cognitive demands than complex WM tasks (e.g., N-back). Hence, span tasks are suitable for measuring WM capacity/span without significant additional cognitive load while minimising the burden on patients and reducing the measurement of other cognitive functions.

Despite the high prevalence of cognitive impairment after a stroke, studies focusing on WM span represent a smaller proportion of the growing body of this research area [2, 17]. Including such studies would be highly recommended, as the integrity of the WM span is essential for other complex WM-related cognitive functions. Consequently, the targeting of these domains raises further questions, and choosing the appropriate cognitive training tools within cognitive rehabilitation presents an additional challenge for professionals.

Over the past few decades, a wide array of cognitive rehabilitation (CR) tools has emerged, encompassing conventional techniques (e.g., paper/pencil exercises), non-invasive brain stimulation (e.g., transcranial magnetic stimulation or transcranial direct current stimulation (tDCS)), and computer-assisted cognitive rehabilitation (CACR), such as computerised cognitive training programs (CCT). Other restorative interventions (e.g., aerobic exercises, pharmacological and educational interventions) are also available [18–21]. An essential aspect of CR is the accessibility and usability of the chosen rehabilitation method. In this regard, CCTs and tDCS are appropriate and popular choices in post-stroke CR, given their low resource intensity, cost-effectiveness and high level of technical support compared to other methods (e.g. TMS). Furthermore, they can be applied in combination to increase their effectiveness [22, 23].

CCT and tDCS exert distinct effects on modulating cognitive function. CCTs offer training possibilities for post-stroke CR and have the potential to facilitate restorative training for cognitive functions by stimulating various cognitive domains, positively influencing patient motivation [24]. As a result, we can expect significant improvement in the domain related to the trained task (near transfer effect), albeit some tasks also involve improvement in non-trained domains (far transfer effect) [25]. Thus, WM improvements can be achieved through targeted and non-targeted CCTs. Various CCTs are currently available, and the choice depends on the preferences and expertise of the training professional. In recent years, recommendations have emerged regarding which CCTs could suit various neurological diseases; for instance, based on Maggio and colleagues’ (2023) research, ERICA and Lumosity can offer appropriate options for post-stroke WM training. In terms of post-stroke CR research, Zhao and colleagues (2021) conclude that computer-assisted training has no significant effect on WM; however, these results are not specific to the WM span. Other studies have found a transfer effect for WM-specific measures, but these have not focused on the WM span either [26].

Conversely, tDCS is a non-invasive neuromodulation technique that utilises direct current to influence sodium-calcium channels and N-methyl-D-aspartate receptors, resulting in alterations to the resting membrane potential [27]. Consequently, a specific cognitive function can be improved through increased neural responsiveness in related brain areas during facilitation; however, this outcome is not universally observed. Various parameters, such as dosing, intensity, and duration, can result in reversing effects if they are prolonged or not appropriately calibrated. Therefore, it is crucial for specialists to carefully adjust these parameters to ensure optimal outcomes [28]. Studies on WM training primarily focus on the prefrontal and dorsolateral prefrontal cortex (DLPFC); recent research has also emphasised the importance of a broader frontoparietal network [29–33]. The effectiveness of tDCS stimulation is influenced by various settings, such as stimulation strength or location. Neurological conditions such as stroke can modify normal responses in neuronal activity, necessitating consideration in the training approach for practitioners [34–36]. The use of tDCS alonein post-stroke WM research is less pronounced; tDCS is usually utilised in combination with CCT. Hence, the effect is challenging to interpret.

This systematic review and meta-analysis aims to understand the effects of two widely-used and cost-effective CR procedures, CCTs and tDCS, on WM span in post-stroke individuals. The mechanisms of these techniques are different and partly unclear, with the potential for improving specific aspects of WM yet to be explored. Our study focused on comparing RCTs, deliberately excluding combined applications of these techniques. We specifically targeted the assessment of WM span, comparing tests used in clinical practice, such as the Digit Span Forward, Digit Span Backward, and Visual Span Tests, as outcome measures. This analysis represents one of the first attempts to explore the effects of these rehabilitation techniques on WM span in post-stroke individuals, providing insights into their potential efficacy and mechanisms of action.

## Methods

The present meta-analysis aimed at synthesising the results of controlled studies on the rehabilitation effects of CCT and tDCS on WM span in post-stroke individuals. The meta-analysis was conducted according to PRISMA (Preferred Reporting of Systematic Reviews and Meta-Analyses) standards with a PROSPERO registration protocol (ID: CRD42023387182) [37]. The review protocol and data used in the analysis are available from the authors (for the PRISMA 2020 Checklist, see Supplementary Materials).

### Eligibility criteria

According to the PICOS model, the inclusion/exclusion criteria are presented in Table 1.

**Table 1:** Inclusion/Exclusion criteria according to PICOS model

	Include	Exclude
Population	Persons with average age between 40.0 and 70.0 in post-stroke condition.	Persons younger than 40.0 or older than 70.0 years of age on group average
		Persons with other/comorbid neurological conditions
Intervention(s)	CCT or tDCS with additional rehabilitation treatment	Combined application of CCT and tDCS
Comparison	Control group as conventional CR techniques or other restorative interventions or sham condition (for tDCS) or passive/waiting list control	Control group as healthy control or having neurological conditions other than stroke.
		tDCS control group receiving CCT/CACR.
Outcomes	At least one outcome falls in the group of the following measures: Digit Span Forward Task, Digit Span Backward Task, Visual Span Task, Corsi Block Tapping Task	None
Study designs	Studies including pre or/and post-intervention data	Not randomized and/or controlled studies
	Randomized and controlled studies, pilot studies were acceptable	
***Inclusion/Exclusion criteria of publication characteristics***		
	**Include**	**Exclude**
Publication types	Primary empirical studies	Non primary empirical studies including opinions, discussions or editorials
	Published or in-press studies	
	Working papers of empirical studies	Reviews, meta-analyses.
Years of publication	2000 -	Studies before 2000
Language	English language	Non-English language

### Outcome measures of working memory span

In the selection of outcome measures, we aimed to select WM measures that (a) are relevant and commonly used in clinical practice, (b) measure WM span/capacity with the lowest possible additional cognitive load, and (c) measure either verbal or visual modalities *(detailed justification for these criteria can be found in theBackgroundsection).* Based on theoretical considerations and previous research, the following instruments meet these criteria:

#### Short-term verbal recall - digit span forward test (DSTF)

The task requires the subject to recall sequences of numbers of different lengths in the order in which the examiner tells them. The capacity usually ranges from 3 to 9 units, during which no manipulation of the spoken information is required. The most widely used neuropsychological test procedure for this purpose is the Digit Span Test, part of the Wechsler Intelligence Test [38].

#### Working memory - digit span backward test (DSTB)

A procedure for testing WM in which the subject is asked to recall a series of numbers of different lengths in reverse order. The task involves retaining and manipulating acquired information and integrating CE and short-term memory capacity. The most widely used neuropsychological testing procedure for this purpose is the Digit Span Backward Test as a part of the Wechsler Intelligence Test [38].

#### Visual working memory – visual span test/corsi block tapping task (VSTF)

Visual WM (visuospatial sketchpad) is usually measured using the Visual Span Test or Corsi Block Tapping Task. In this test, the subject is shown several sequences of a growing number of visual elements in a specified order (e.g., blocks up to 9), which they must reproduce in the same series [39].

### Search strategy

Two authors (Cs.K. and K.B.) performed systematic searches in PubMed, Scopus, Cochrane Library and Embase databases, complemented by a manual search of studies between 15/12/2022 and 10/01/2023. The search strategy applied an extended combination of keywords related to the PICOS, with particular focus on WM and related measures (‘working memory’ or ‘executive functions’ or ‘cognition’ or ‘memory’ or ‘digit span’ or ‘short-term memory’ or ‘visual span’ or ‘spatial span’ or ‘Corsi block tapping task’) and (‘tdcs’ or ‘transcranial direct current stimulation’ or ‘cognitive training’ or ‘computer-based cognitive training’ or ‘computer-assisted cognitive training’ or ‘cct’ or ‘computerised cognitive training’ or ‘computerised cognitive training’) and (‘stroke’ or ‘post-stroke’ or ‘patients after stroke’ or ‘post-stroke patients’). Two authors (Cs.K. and K.B.) independently created the database and screened titles and abstracts for inclusion criteria. We identified further potential studies by reviewing references related to the topic.

### Study selection

**Fig. 1:**
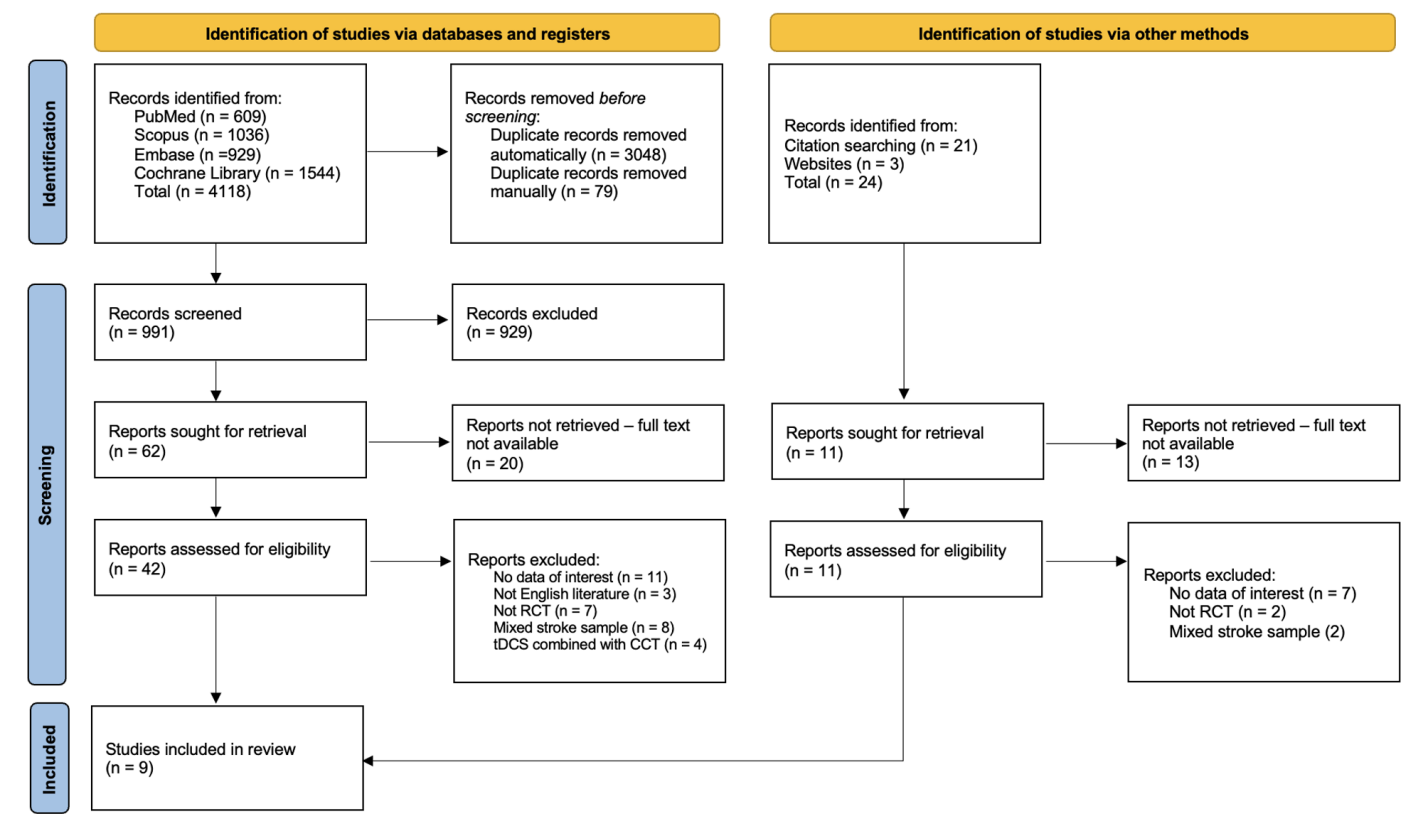
Preferred reporting items for systematic reviews and meta-analyses (PRISMA) flow diagram of study selection

Two authors (Cs.K. and K.B.) independently reviewed which articles were to be included in the analysis. After building the databases, the collected references were imported into the Endnote 20.3 (Clarivate) reference management program. The process was then carried out as follows: (1) duplicates were first automatically and then manually deleted; (2) the titles were filtered manually, deleting articles containing terms that did not meet the PICOS criteria (e.g., traumatic brain injury, Parkinson’s disease, transcranial magnetic stimulation); (3) the abstracts were filtered according to the same rule; (4) the full texts of the remaining articles were reviewed; (5) if it was not possible to collect at least five relevant studies for an intervention, the meta-analysis would not be carried out. The final decisions regarding the inclusion and exclusion of studies were compared between the two reviewers. The references of the included records were automatically and manually screened, with the task divided between the two reviewers. The interrater agreement between the two reviewers was assessed using Cohen’s kappa statistic. Cohen’s Kappa was calculated for the initial search, resulting in a value of 0.71, indicating substantial agreement. However, the combination of the neuropsychological tests and interventions we investigated may result in rare events within the selected population; we calculated the Prevalence-Adjusted and Bias-Adjusted Kappa (PABAK) to provide a more accurate measure of agreement. The PABAK was calculated for both the abstract and full-text screening phases based on the work of Mackinnon (2000) [40]. Based on the results, the agreement between the two reviewers was adequate (Abstract: Cohen’s Kappa = 0.75, PABAK = 0.79; Full-text: Cohen’s Kappa = 0.77, PABAK = 0.82). Any discrepancies were resolved by discussion. We used the flowchart adopted by PRISMA to illustrate the selection process visually (Fig. 1).

### Risk and bias assessment

The methodological quality of each article included was independently and critically assessed by two authors (Cs.K. and K.K.) using Version 2 of the Cochrane Risk of Bias Tool (RoB2), which allowed the analysis of six domains: (1) randomisation process (2) deviation from intended interventions, (3) missing outcome data, (4) measurement of the outcome, (5) selection of the reported result, (6) overall bias [41]. Each domain was classified as ‘low’, ‘high’ or ‘some concerns’.

### Data extraction

Only data containing the appropriate population, interventions, comparisons, outcome variables, and research design were extracted by two authors (K.Cs. and K.B). Post-intervention means, standard deviations, medians and IQR were collected during data extraction. Descriptive information included: author and year, number of subjects, mean age, sex distribution, type of stroke, time after stroke, type of intervention, duration of intervention (minutes/sessions), type of control and primary outcomes. Stimulation location, dose, electrode size, and current density were listed for tDCS studies. Where two or more groups of interventions were included in the same study, they were interpreted separately according to the recommendations of the Cochrane Handbook [41]; furthermore, in the case of more than one control group (e.g. active control vs. waiting list control), we interpreted active controls. Discrepancies identified during cross-checking were resolved through discussions. In the case of Wentink and colleagues (2016), this involved converting median and interquartile range values (IQR) into means and standard deviations (SD) for subsequent statistical calculations.

### Statistical analysis and meta-regression

The meta-analysis used Review Manager Software Desktop and Web (RevMan, version 5.4). Post-intervention means, standard deviations (Mean ± SD) and variances were collected from each selected study as post-intervention data for the outcome in the experimental and control groups. In the case of non-normal data, a transformation was performed into mean and ± SD, using the given medians and IQR values based on previous methodological recommendations [42, 43]. For heterogeneity, I^2^ and P-values were determined and considered significant if *P* < 0.10 or I^2^ > 50%. Values for I^2^ are expressed as a percentage, with suggested values of 25% (low), 50% (moderate), and 75% (high) used to categorise levels of heterogeneity [44]. The analysis converted means and standard deviations to standardised mean differences (SMD) with a 95% confidence interval (CI). A random-effects model was used in every case since the collected studies showed moderate variability from the viewpoint of intervention and measurement type [45]. We assessed each study’s effect on the overall effect size to test sensitivity, and removed studies individually responsible for heterogeneity [46]. Based on previous methodological considerations, we classified effect sizes into 0.2 (small), 0.5 (medium) and 0.8 (large) categories [47]. The indication of statistical significance was set at *P* < 0.05. In the analysis performed, heterogeneity is expected to emerge, presumably between-study differences that may appear in the studies regarding training time and mean age. To explore this, we planned to run a meta-regression for the following continuous variables: age (AGE), number of interventions (NO. OF SESSIONS), and time per intervention (DURATION), using a random-effects model with the Restricted Maximum Likelihood (REML) method in the Jamovi project (Package for R, 2018, Jamovi project, Version 0.9). The random effects model accounts for variability within and between studies by including random effects, thus accommodating heterogeneity among study results. To accurately estimate the variance components of this model, we employed REML, which is particularly suited for this purpose as it provides less biased estimates of the between-study variance (τ²) by focusing on the likelihood of the residuals after accounting for the fixed effects. According to recent studies [48], REML reduces bias in estimating variance components and improves the accuracy of heterogeneity assessment, making it a preferred method in meta-analysis. This approach ensures robust and precise heterogeneity estimation, thereby enhancing the reliability of our meta-regression findings.

## Results

### Search results

Initially, 4118 articles were identified from the electronic databases (PubMed: 609; Scopus: 1036; Embase: 929; Cochrane Library: 1544), with 24 additional publications identified during manual search, resulting in 4142 articles in total. This was followed by excluding duplicates, manual filtering, and screening of titles and abstracts, narrowing the preliminary extensive database to 53 publications. Subsequently, further 44 studies were filtered out as they were not in English (3), not performed on purely stroke population (10), not RCTs (9), did not include the previously defined outcome variables or did not meet the inclusion criteria (18), or tDCS was used in combination with CCT (4) (Fig. 1). Finally, nine articles were included in the study, all of which used CCT solely [19, 43, 49–55] and no study could be selected for tDCS intervention to apply a meta-analysis (Fig. 1).

### Results of risk and bias assessment

**Fig. 2:**
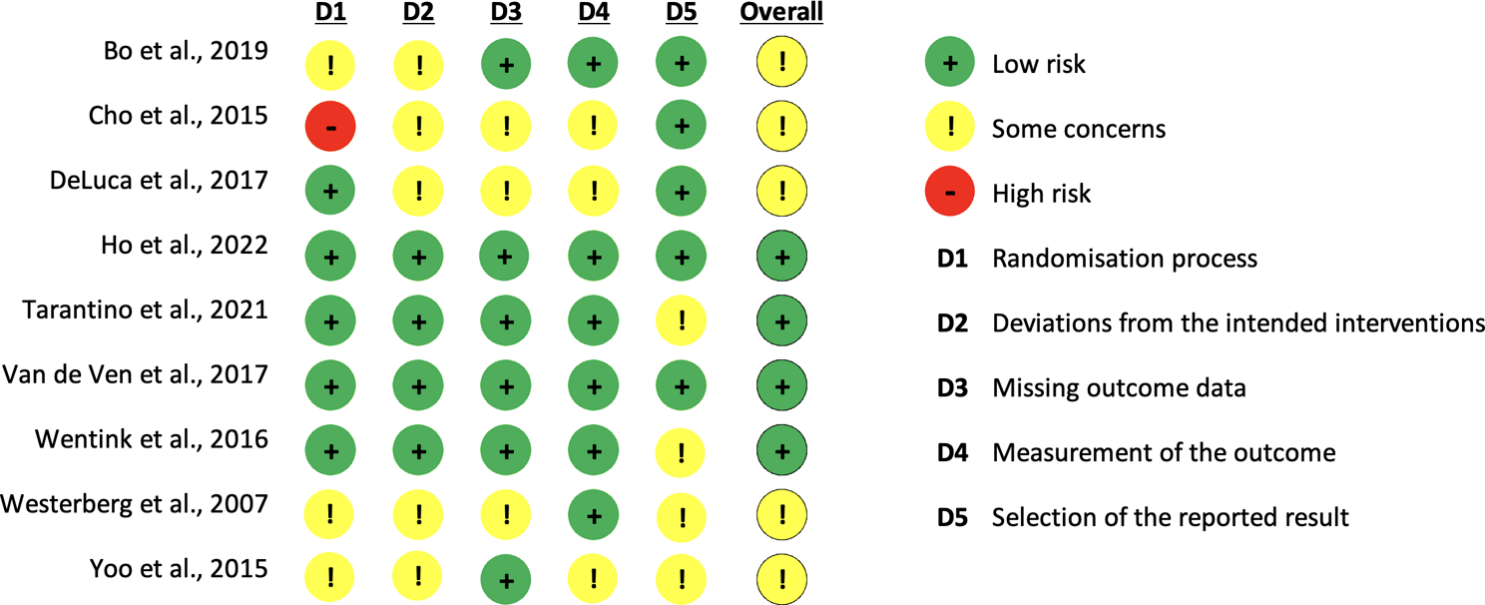
Results of risk and bias assessment according to the cochrane risk of bias tool

Four selected articles were classified as ‘low-risk’, five as ‘some concern’, and none as ‘high risk’. Concerning the randomisation and allocation, we could not entirely assess the process used in one case [19]. In four cases, several factors raised questions about randomisation or allocation In the other cases, the studies met this requirement [19, 49, 54–55]. Furthermore, in three studies, the choice of measurement instruments was not clearly justified [19, 50, 55]. The studies targeted global cognition in several cases. Thus, we could not identify an apparent risk factor for WM-related measurements we chose in our analysis. The details of the methodological quality assessment are shown in Fig. 2.

### Study characteristics

The articles were published between 2007 and 2022. A total of 461 (Intervention: 234 Control: 227) subjects were included in CCT studies. The countries involved were China (1), Italy (2), Korea (2), Netherlands (2), Sweden (1), and Taiwan (1). The studies showed a wide range of sample sizes, mean ages, and sex distributions, focusing on various stroke types and cognitive impairments. Sample sizes across studies varied, with intervention groups ranging from 9 to 50 (Mean: 25) participants and control groups from 9 to 57 (Mean: 26) participants. The range of age spanned from 43.9 to 67.5 years (Mean: 59.0), with a mixed distribution of males and females (M/F ratio: 1.31:1). In the selected studies, we found a mixture of primarily ischemic stroke (IS) and intracerebral haemorrhage (ICH) (5 studies) and vascular cognitive impairment (VCI) (1 study), while in three cases, data on medical background/condition/aetiology was not included. The average time post-stroke among participants ranged from 3 months to 28.3 months (Mean: 13.8). The studies included five different types of control conditions. These control conditions were utilised as follows: “Other restorative intervention” was employed in 4 studies, “Conventional Cognitive Rehabilitation” was used in 2 studies, “Conventional CR and Other Restorative Intervention” was applied in 1 study, “Waiting List Control” was used in 1 study, and “Passive Control” was employed once. The primary aims across these studies were to improve various cognitive functions such as “general cognitive functions” were the primary aim in 5 studies, “memory and attention” was the focus in 1 study, “executive functions” was targeted in one 1 study, “cognitive flexibility” was the primary aim in 1 study, and “working memory” was the focus in 1 study (for study characteristics, see Table 2).

**Table 2:** Characteristics of CCT and tDCS studies

Author/Year	Sample size (Intervention/Control)	Mean Age (years)	Sex (M/F)	Stroke Type	Time after stroke (avg. months)	Additional treatment to CCT	Type	Duration (Mins/Sessions)	Control	Aim/primary outcomes
Bo et al. [49]	45/47	67.50	21/24	VCI	< 6.0	Physical exercise	COGPACK	60 min/ 36 sessions	Other restorative intervention	Cognitive functions / DSTF, TMT-B, Stroop, Mental Rotation
Cho et al. [19]	12/13	60.00	7/5	N/A	5.3	Other restorative intervention	RehaCom	30 min/ 24 sessions	Other restorative intervention	Memory and attention / DSTF, DSTB, VSTF, VCPT, ACCPT
De Luca et al. [50]	20/15	43.90	11/9	IS/ICH	3.0	Conventional CR	Erica	45 min/ 24 sessions	Conventional CR	Cognitive functions / DSTF, BNT, MMSE, PVF, RAV, RAVL-I/R, SVF, TT
Ho et al. [51]	19/20	63.60	12/7	IS/ICH	19.9	Conventional CR	Lumosity	20 min/ 24 sessions	Conventional CR	Cognitive functions / DSTF, DSTB, VSTF, MMSE, SDMT
Tarantino et al. [52]	18/19	64.60	12/6	IS/ICH	3.1	Conventional CR and other restorative intervention	Pilot with E-prime	60 min/10 sessions	Conventional CR and Other restorative intervention	Executive functions / DSTF, DSTB, VSTF, TMT-A, PVF, SVF, WCST, Stroop
Van de Ven et al. [53]	38/24	60.90	19/16	N/A	28.3	Conventional CR and other restorative intervention	BrainGymmer	30 min/ 58 sessions	Waiting list control	Cognitive flexibility / VSTF, TMT-A/B, DSTF, RAVL, N-back, Raven PM
Wentink et al. [43]	50/57	59.00	34/19	IS/ICH	26.0	No	Lumosity	20 min/ 40 sessions	Other restorative intervention	Cognitive functions / DSTF, DSTB, TMT-A/B, Flanker Task, Raven PM
Westerberg et al. [54]	9/9	55.00	8/1	IS/ICH	20.8	No	RoboMemo	40 min/ 23 sessions	Passive control	Working memory / DSTF, VSTF, Stroop, Raven, PASAT, Ruff 2 × 7
Yoo et al. [55]	23/23	56.30	9/14	N/A	11.8	Other restorative intervention	RehaCom	30 min/ 25 sessions	Other restorative intervention	Cognitive function / DSTF, VSTF, VeLT, ViLT, ACCPT, VCPT, TMT-A

*Abbreviations* Trail-Making Test (TMT), Stroop Test, Mental Rotation, Visual Continuous Performance Test (VCPT); Auditory Controlled Continuous Performance Test (ACCPT), Boston Naming Test (BNT), Mini-Mental State Examination (MMSE), Phonemic Verbal Fluency (PVF); Raven’s Coloured Progressive Matrices (RAV); Rey Auditory Verbal Learning Test Immediate (RAVLI); Rey Auditory Verbal Learning Test Late (RAVLL); Semantic Verbal Fluency (SVF); Token Test (TT); Symbol Digit Modalities Test (SDMT), Wisconsin Card Sorting Test (WCST), Paced Auditory Serial Addition Test (PASAT), Verbal Learning Test (VeLT), Visual Learning Test (ViLT), Digit Span Forward Test (DSTF), Digit Span Backward Test (DSTB), Visual Span Forward Test (VSTF)

### Intervention characteristics

We found six different CCTs (Cogpack, RehaCom, Erica, Lumosity, RoboMemo, BrainGymmer) and, in one case, results from an unknown, non-standardized rehabilitation program. The CCTs were generally used either alone or in addition to traditional enhancement program, with a duration of 20 to 60 min across sessions ranging from 10 to 58. Lumosity (www.lumosity.com) and RehaCom (www.hasomed.de/en/products/rehacom/), software were the most commonly used for the selected studies, offering 30 modules depending on the specified functions. Furthermore, Cogpack (www.markersoftware.com) offers 64 tasks, such as visual-motor integration, learning, memory, attention and EF. Cogpack is widely used in clinical settings as the functions can flexibly adapt to individual needs. RehaCom offers more than 30 training modules to improve memory, attention, executive functions and visuospatial skills. It is often used in neurorehabilitation for patients with neurocognitive impairment following stroke or traumatic brain injury. RehaCom allows the difficulty of the tasks to be adjusted in real-time based on the patient’s performance, thus providing personalised cognitive training. Erica (www.erica.giunti.it), has customizable modifications for five cognitive domains; the program is primarily known for its user-friendly interface and ability to create customised cognitive training plans. Lumosity includes over 50 training exercises to improve memory, attention, problem-solving, and mental flexibility. It is available via a web browser and mobile platform; hence, patients can easily integrate cognitive training into their daily routine. RoboMemo (www.cogmed.com). includes visuospatial and auditory WM tasks that target specific areas of cognitive functioning. Previous studies have shown that RoboMemo can significantly improve WM capacity. BrainGymmer (www.braingymmer.com) includes a variety of cognitive tasks to improve attention, inhibitory control, cognitive flexibility and WM. It offers a wide range of motivational tasks with a user-friendly design. BrainGymmer also provides feedback and progress monitoring.

### Outcome characteristics

As for neuropsychological tests, all of the selected studies included at least one measure of DSTF, DSTB and VSTF, in addition to the following tests to measure cognitive function: Trail-Making Test (TMT), Stroop Test, Mental Rotation, Visual Continuous Performance Test (VCPT); Auditory Controlled Continuous Performance Test (ACCPT), Boston Naming Test (BNT), Mini-Mental State Examination (MMSE), Phonemic Verbal Fluency (PVF); Raven’s Coloured Progressive Matrices (RAV); Rey Auditory Verbal Learning Test Immediate (RAVLI); Rey Auditory Verbal Learning Test Late (RAVLL); Semantic Verbal Fluency (SVF); Token Test (TT); Symbol Digit Modalities Test (SDMT), Wisconsin Card Sorting Test (WCST), N-back, Flanker Task, Paced Auditory Serial Addition Test (PASAT), RUFF 2&7, Verbal Learning Test (VeLT), Visual Learning Test (ViLT). Regarding the rehabilitation effect, significant improvement in cognitive functions was found in 8 cases (88%) in CCT studies.

### Meta-analysis outcomes

Since the evaluation methods were inconsistent, the standard mean difference method was chosen for analysis. Furthermore, we applied the random-effects model due to the variability of study parameters. Short-term recall (DSTF) was analysed in eight studies involving CCT (*n* = 384). Heterogeneity was moderate (*P* = 0.05; I2 = 50%), and the meta-analysis showed no significant improvement compared to the control group (Z = 1.95; *P* = 0.05) (SMD = 0.30, 95%CI = 0.06 to 0.46, I^2^ = 50%). According to the funnel plot analysis, we identified asymmetry: the sensitivity analysis results indicated that two studies (Bo et al., 2019; Westerberg, 2007) have contributed to heterogeneity. After removing these studies, heterogeneity was reduced considerably; however, still no significant difference occurred (*P* = 0.54, I^2^ = 0%) (SMD = 0.08, 95% CI = -0.15 to 0.32; Z = 0.70; *P* = 0.49).

Four CCTs (*n* = 193) were analysed with WM (DSTB) as an outcome, in the case of which the analysis showed significant improvement compared to the control group, with low heterogeneity (*P* = 0.93; I2 = 0%) (SMD = 0.39, 95% CI = 0.10 to 0.67; Z = 2.65, *P* = 0.008).

**Fig. 3:**
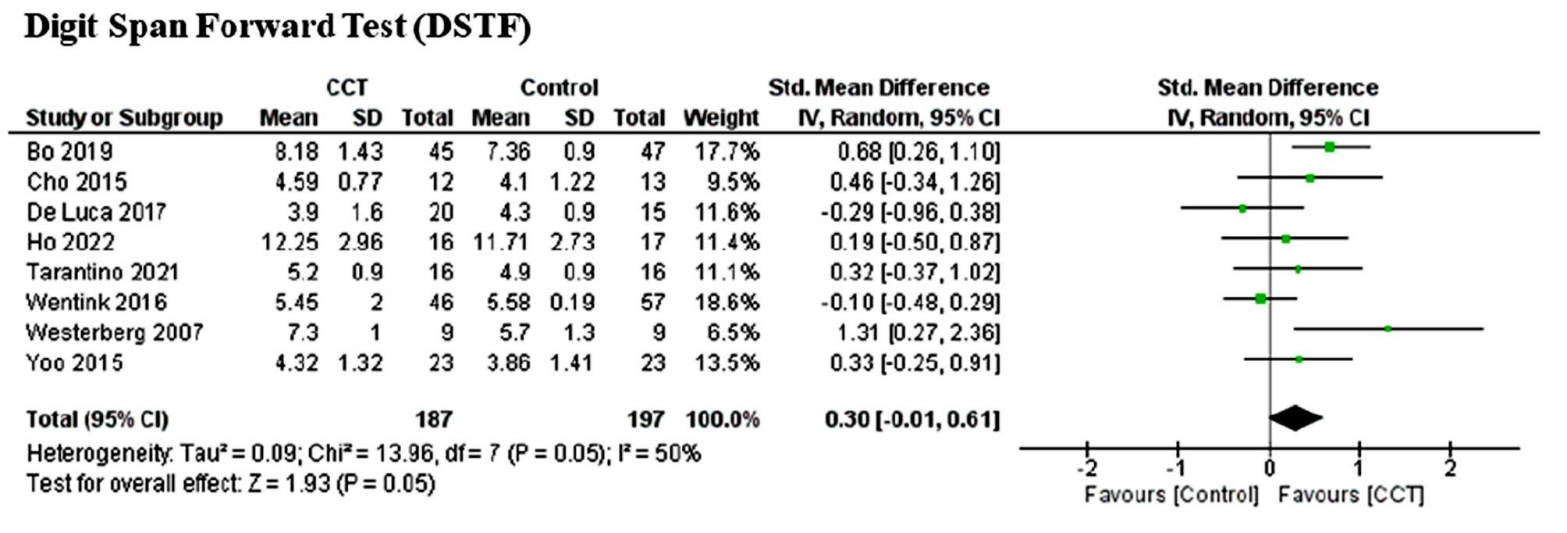
Effect of CCT on short-term recall measured by digit span forward test (DSTF) compared to controls

**Fig. 4:**
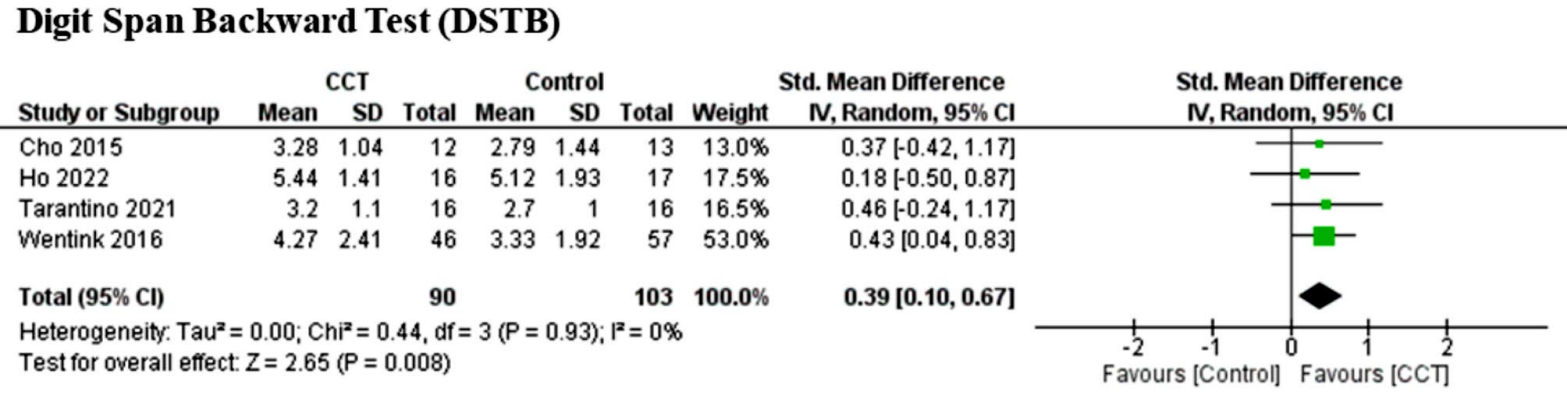
Effect of CCT on WM measured by digit span backward test (DSTB) compared to controls

**Fig. 5:**
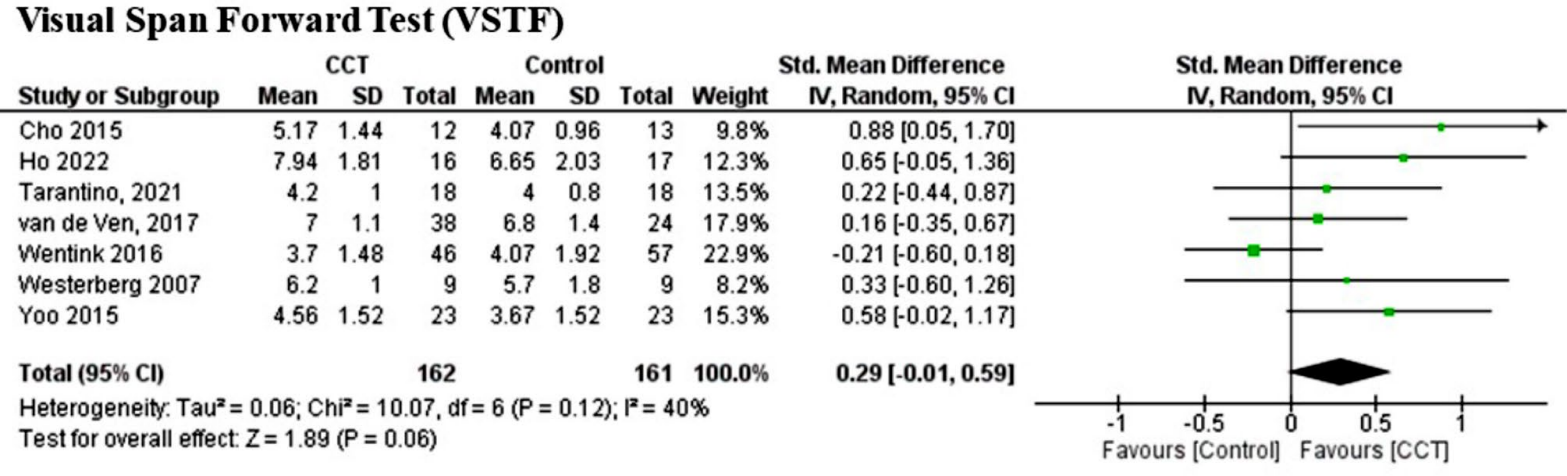
Effect of CCT on visuospatial WM measured by visual span forward test (VSTF) compared to controls

For visuospatial span (VSTF), we found seven studies using a CCT intervention (*n* = 323). The CCT intervention showed no significant difference, with moderate heterogeneity (*P* = 0.12; I^2^ = 40%) (SMD = 0.22, 95%CI = -0.01 to 0.44; Z = 1,90, *P* = 0.06). Based on funnel plot analysis, one research was responsible for moderately strong heterogeneity for CCT (Wentink, 2016). After removing this study, the heterogeneity dropped to zero, and we observed a significant effect compared to the control group (Z = 3.14; *P* = 0.002) (SMD = 0.43, 95%CI = 0.16 to 0.69, I^2^ = 0%) (Figs. 3, 4 and 5; Table 2, 3).

**Table 3:** Results of sensitivity analysis

Outcome	Int.	Before analysis					Removed studies.
		SDM	95% IC	Z/P (Effect)	I^2^	No. S.	
DSTF	CCT	0.30	[-0.01, 0.61]	1.93/0.05	50%	8	Bo (2019), Westerberg (2017)
VSTF	CCT	0.29	[-0.01, 0.59]	1.89/0.06	40%	7	Wentink (2016)
		After analysis					
DSTF	CCT	0.08	[-0.15, 0.32]	0.70/0.47	0%	6	
VSTF	CCT	0.42	[0.15, 0.69]	3.05/0.002	0%	6	

*Abbreviations* Digit Span Forward Test (DSTF), Visual Span Forward Test (VSTF)

### Meta-regression outcomes

In the meta-regression model, the following variables which may cause significant variability for CCT and tDCS interventions were included : ’age’, ’time intervention (mins)’ and ’number of intervention sessions’. Based on the meta-regression results, no significant moderating effect of any variable was found on neuropsychological test results (Supplementary Table 1).

## Discussion

In this study, we examined the effectiveness of CCT and tDCS on WM span, utilising measures including the Digit Span Forward Test, Digit Span Backward Test, and Visual Span Forward Test in individuals post-stroke. The results of our meta-analysis indicate a significant effect of CCT on WM span measured by DSTB and VSTF. However, not enough tDCS studies were available to be included in the analysis. In light of these findings, several points warrant discussion, including the implications of post-stroke CR, the mechanisms underlying the observed effects, and possibilities for future research.

Firstly, the observed improvements in DSTB and VSTF due to CCT interventions highlight the potential of CCT in enhancing WM span after a stroke. These findings align with previous literature suggesting that targeted CCTs can improve general cognitive function. However, it supplements the findings of Van de Ven and colleagues (2016), who found that CCT can be effective on DSTB, but not in VSTF and contradicts the conclusions of Zhao and colleagues (2021), who reported that computer-assisted programs in post-stroke cognitive rehabilitation do not improve WM function. The efficacy of CCT in improving WM can be attributed to several mechanisms; CCT tasks are designed to target specific cognitive processes, such as attention, processing speed, and executive functions, which are known to contribute to WM performance [56]. Through repeated practice and feedback, individuals may develop more efficient cognitive strategies and neural networks supporting WM function [57]. Accordingly, there is no clear evidence that WM tasks, in general, cannot be effectively improved in post-stroke patients. Nevertheless, it is essential to note that in the study of Zhao and colleagues (2021), the subdomain of WM was not specified, as in our case.

Secondly, the heterogeneity of the studies included in our analysis was relatively high. Consequently, interpreting the results should consider variations in study design, participant characteristics, intervention protocols, and outcome measures. For example, Cho and colleagues (2015), Yoo and colleagues (2015), and Ho and colleagues (2022) used a rigorous RCT design, while Bo and colleagues (2019) used a single-blind RCT framework. There were also some differences in control, as Van de Ven and colleagues (2017) used a wait-list control where participants received a delayed intervention. In contrast, Westerberg and colleagues (2007) used a passive control group design in which the control group had no other additional treatment. Tarantino and colleagues (2021) used traditional cognitive rehabilitation and other therapeutic interventions as controls. Participant characteristics also differed between studies; participants ranged in age from 43.9 to 64.6 years. The time since stroke also varied, with some studies recruiting participants within 3–6 months after stroke (e.g. Cho et al., 2015) and others recruiting participants within a longer period after stroke, such as 28.3 months, as in the study by Van de Ven and colleagues (2017). These differences between age and time since stroke can significantly affect the outcomes of interventions; for example, a study by Knoflach and colleagues (2012) found that younger stroke patients tend to have better rehabilitation outcomes than older patients [58]. This highlights the importance of considering patient demographics and stroke chronology when evaluating the effectiveness of rehabilitation interventions.

Regarding rehabilitation programmes, there was considerable heterogeneity in the intervention protocols used in the studies. For example, Yoo and colleagues (2015) used the RehaCom software for 30 min per session five times a week for five weeks. In contrast, Cho and colleagues (2015) used a six-week training protocol with two 30-minute sessions per week. Bo and colleagues (2019) combined physical activity and cognitive training in structured 12-week-long, 50-60-minute sessions three times a week. Different intervention protocols may result in variations in the intensity and duration of CCTs, which are vital components for neuroplasticity [59]. Furthermore, studies have shown that higher intensity and longer duration of CCTs are associated with better outcomes in improving cognitive function; however, the individual abilities of patients should be taken into account to avoid unnecessary strain [60].

The neuropsychological tests used to assess additional cognitive functions also varied widely; however, the measures we selected were available in all test sets. This may be significant because different studies may have targeted different cognitive functions. For example, Yoo and colleagues (2015) and Cho and colleagues (2015) used digit span tasks (both forward and backward) and visual range tests to assess WM. Bo and colleagues (2019) used a broader range of cognitive tests, including the Trail Making Test Part B, the Stroop Test, the DSFT, and mental rotation tasks to assess cognitive flexibility and selective attention. Considering these differences in measurement may be crucial in post-stroke research, as they may significantly affect the comparability and interpretation of results. Different neuropsychological testing procedures may focus on different aspects of cognitive function, which may lead to differences in reported results. Standardisation of outcome measures in post-stroke cognitive rehabilitation research would ensure consistency and reliability in assessing the effectiveness of interventions; furthermore, despite heterogeneity, we found measurable effects on our selected neuropsychological tests, demonstrating the robustness and potential efficacy of interventions in different settings.

In summary, based on the broader literature, heterogeneity determinants such as study design, participant characteristics, intervention protocols, and variation in outcome measures can significantly impact the results and their interpretation. Likewise, the type and intensity of cognitive training may also affect the results. However, despite the significant heterogeneity, we found measurable effects on our selected neuropsychological tests, indicating the robustness and potential effectiveness of the interventions. Yet, the variables examined in the meta-regression (e.g. age, duration, no. of sessions) did not prove to be significant factors.

Our results can also be interpreted through Baddeley’s WM model, according to which WM consists of several components: the phonological loop, the spatial-visual sketchpad, the episodic buffer and the CE. The improvements observed in DSBT suggest improvements in the phonological loop and CE, as this task involves verbal WM and complex information manipulation. Similarly, the improvements observed in VSFT indicate an improvement in the spatial-visual sketchpad. The lack of improvement seen in DSFT, primarily related to the phonological loop, suggests that training programs are more effective in improving tasks requiring more complex cognitive processing than simple memory tasks. However, this phenomenon can be explained by the research of Donolato and colleagues (2017), who found significant differences between forward and backward versions of verbal WM tasks, as the backward version typically represents a higher cognitive load for the participants in their study. However, no such differences between forward and backward recall tasks were found for the spatial-visual sketchpad in healthy individuals [61], suggesting no significant difference in CE load for the visual modality, but there is for the verbal modality. These results indicate that CCT can effectively target and enhance specific elements of WM, particularly those involving more complex manipulation of information.

The question emerges whether targeted cognitive training designed to improve WM could yield significant effects on general cognitive function. More specifically, CCT interventions may promote neuroplasticity and facilitate the recruitment of neural networks associated with near or far transfer effects [62]. Our study specifically collected WM span measures with the lowest possible cognitive load to illustrate the training potential of low-load WM processes. Notably, the possible far transfer effects observed in our study suggest that improvements in trained tasks may generalise to untrained tasks, underscoring the potential for broader functional gains following CCT interventions. Additionally, our meta-analysis revealed improvements in verbal and visual WM tasks following CCT, suggesting that training effects may generalise across different modalities. Furthermore, the mechanisms underlying the observed improvements in WM span following CCT interventions merit consideration. Neuroimaging studies have implicated a network of brain regions, including the prefrontal cortex and parietal cortex, in WM processes [29]. Future research employing neuroimaging techniques could provide detailed insights into the neural mechanisms mediating the effects of CCT on WM span in post-stroke individuals.

Moreover, although our study looked strictly separately at the effects of tDCS and CCT in post-stroke patients, in many cases, these interventions are used together. Hence, the potential synergistic effects of combining CCT with other rehabilitation modalities warrant investigation. Previous studies have suggested that multimodal interventions combining cognitive training with non-invasive brain stimulation techniques, such as tDCS, may yield enhanced cognitive outcomes compared to single-modality interventions [63, 64]. By targeting complementary neural mechanisms, multimodal interventions can potentially maximise neuroplasticity [63]. Recent studies have shown that combining tDCS and CCT can improve WM outcomes in people with neuropsychiatric disorders [65]. For instance, tDCS can modulate cortical excitability and improve neuroplasticity, thereby potentially enhancing the effects of cognitive training [66]. In healthy populations, the synergistic effects of tDCS and CCT have been investigated with promising results [67]. For example, Andrews and colleagues (2011) found that combining anodal tDCS and WM training led to significant improvements in performance compared to sham stimulation [68]. Similarly, a study by Martin and colleagues (2014) reported enhanced learning rates and retention of trained tasks when tDCS was used alongside CCT. Future research would benefit from exploring the long-term effects of combined tDCS and CCT interventions and should investigate optimal stimulation parameters, including electrode placement, current intensity and session duration. In addition, understanding individual differences in response to these interventions, such as baseline cognitive abilities, may help to develop tailored treatment strategies [69].

Furthermore, the choice of outcome measures in assessing WM span and methodological considerations regarding measurement procedures are pivotal to understanding CR interventions’ impact on WM function. While DSTB and VSTF are commonly utilised in clinical practice, they represent only a subset of the multifaceted cognitive processes within the WM domain. Future studies could benefit from employing a broader range of WM tasks, encompassing verbal and visuospatial span measures and tasks assessing executive functions such as updating, shifting, and inhibition [4]. Research often leans towards employing WM tasks with higher loads, such as N-back, Stroop, and Flanker tasks. Additionally, current research commonly utilises ‘N-back’ tasks as indicators, raising questions about the role of the CE in short-term verbal recall and visual WM. Using DSTB test results as indicators, we measured a different function than ‘N-back’ tasks, with complex WM being necessary for both but not equivalent between the two [13]. While DSTB may emphasise capacity, ‘N-back’ tasks place greater emphasis on specific CE functions, mainly updating. This underscores the impact of CCT on WM function, even without specific training modules.

In contrast to the positive effects observed for CCT, our meta-analysis did not find sufficient evidence to support the efficacy of tDCS in improving WM in post-stroke individuals. This finding is still surprising, given the growing interest in tDCS as a potential intervention for CR [66]. However, it is essential to note that the tDCS literature in post-stroke populations is relatively limited, and studies vary widely regarding stimulation protocols, outcome measures, and participant characteristics. Most of the tDCS research uses it as an adjunctive neuromodulation tool, and there are few available randomized controlled trials of its inedependent use. Furthermore, the variability in stimulation parameters, such as electrode placement, current intensity, duration, and place of stimulation, influence treatment outcomes [70, 71]. Individual differences in neuroplasticity, lesion location, and stroke severity may have influenced responsiveness to tDCS [72]. Future research should aim to elucidate the specific effects of tDCS on WM span in stroke survivors, considering factors such as stimulation parameters, target brain regions, and individual differences in response to stimulation.

Some further general limitations indicate that the current results should be treated cautiously. First, for CCT, the high variability and unclear focus in research settings make it difficult to interpret the results specifically for WM capacity. To address and reduce heterogeneity, future research should consider certain methodological improvements: (1) Establishing standardized protocols for cognitive training interventions, including the duration, frequency, and type of training. (2) Adopting a core set of standardized cognitive assessments widely accepted and validated in stroke rehabilitation research may improve the consistency of outcome measurements. (3) Providing comprehensive details about participant demographics, stroke severity, and time since stroke may help contextualize the findings and enable better comparisons between studies. (4) Conducting long-term follow-up assessments to evaluate the sustainability of cognitive improvements may provide insights into the long-term efficacy of interventions. (5) Further exploring the synergistic effects of combining cognitive training with other rehabilitation modalities, such as tDCS or physical exercise, can shed light on the added benefit of complex intervention in post-stroke cognitive rehabilitation. Following the above suggestions would not only improve the quality of research, but may also allow for more accurate comparisons across studies. Finally, for future practical considerations, there are several factors to consider regarding post-stroke rehabilitation; for instance, the optimal utilisation of financial and personnel resources in the rehabilitation procedure could enhance the benefits of CCT or tDCS.

## Conclusion

In conclusion, our results indicate that CCT interventions are promising in enhancing WM span in post-stroke individuals, as demonstrated by improvements in DSTB and VSTF. However, there remains a need for further investigation into the specific impact of tDCS on WM span in this population. Additionally, future research should delve into the underlying neural mechanisms driving these observed effects and explore the potential synergies between cognitive training and non-invasive brain stimulation interventions. With a more advanced understanding in this field, researchers can guide the development of targeted and efficient rehabilitation strategies to improve cognitive outcomes and enhance the quality of life of post-stroke individuals. Furthermore, our research also points out that the number of RCTs for post-stroke rehabilitation is still low, and there is even less focus on specific studies of WM.

## Electronic supplementary material

Below is the link to the electronic supplementary material.

Supplementary Material 1

## Data Availability

No datasets were generated or analysed during the current study.

## Abbreviations

WM: Working memory
tDCS: Transcranial Direct Current Stimulation
CCT: Computerized cognitive training
EF: Executive functions
CE: Central executive
DSTF: Digit Span Forward Test
DSTB: Digit Span Backward Test
VSTF: Visual Span Forward Test
RCT: Randomized Controlled Trial
DLPFC: Dorsolateral Prefrontal Cortex
CR: Cognitive Rehabilitation
CACR: Computer-Assisted Cognitive Rehabilitation
