# Supplementary material for: The effect of computerized cognitive training and transcranial direct current stimulation on working memory among post-stroke individuals: a systematic review with meta-analysis and meta-regression
Source: BMC Neurol. 2024 Sep 4;24:314. doi: 10.1186/s12883-024-03813-x (PMC11373461; doi:10.1186/s12883-024-03813-x)
Supplement: Supplementary file 1 — Supplementary Material 1 [file 12883_2024_3813_MOESM1_ESM.docx]

| **Supplementary Table 1: Results of meta-regression in the case of CCT** | | | | | |
| --- | --- | --- | --- | --- | --- |
| Moderator | No of comparisons (k) | Z | p | Risk ratio estimate (95% CI) | Tau^2^/Q |
| **CCT** | | | | | |
| **Digit Span Test Forward (DSTF)** | | | | | |
| **Age** | 8 |  |  |  | 0.043/9.911 |
| Intercept |  | 1.24 | 0.217 | -1.441 (3.728 to 0.845) |  |
| Moderator |  | 1.49 | 0.135 | 0.029 (0.009 to 0.067) |  |
| **Duration** | 8 |  |  |  | 0.040/9.692 |
| Intercept |  | 0.40 | 0.692 | -0.129 (-0.767 to 0.509) |  |
| Moderator |  | 1.40 | 0.161 | 0.011 (-0.004 to 0.027) |  |
| **No of sessions** | 8 |  |  |  | 0.109/13.907 |
| Intercept |  | 0.82 | 0.413 | 0.436 (-0.609 to 1.483) |  |
| Moderator |  | 0.26 | 0.796 | 0.005 (-0.040 to 0.031) |  |
|  |  |  |  |  |  |
| **Digit Span Backward Test (DSTB)** | | | | | |
| **Age** | 4 |  |  |  | 0/0.364 |
| Intercept |  | 0.37 | 0.707 | 1.418 (-5.968 to 8.804) |  |
| Moderator |  | 0.27 | 0.748 | -0.017 (-0.138 to 0.104) |  |
| **Duration** | 4 |  |  |  | 0/0.387 |
| Intercept |  | 1.03 | 0.302 | 0.323 (-0.291 to 0.938) |  |
| Moderator |  | 0.23 | 0.819 | 0.002 (-0.017 to 0.022) |  |
| **No of sessions** | 4 |  |  |  | 0/0.424 |
| Intercept |  | 0.78 | 0.434 | 0.336 (-0.506 to 1.178) |  |
| Moderator |  | 0.13 | 0.900 | 0.001 (-0.024 to 0.027) |  |
|  |  |  |  |  |  |
| **Visual Span Forward Test (VSTF)** | | | | | |
| **Age** | 7 |  |  |  | 0.092/10.082 |
| Intercept |  | 0.04 | 0.970 | 0.127 (6.373 to 6.626) |  |
| Moderator |  | 0.05 | 0.957 | 0.002 (0.105 to 0.111) |  |
| **Duration** | 7 |  |  |  | 0.093/9.601 |
| Intercept |  | 0.55 | 0.580 | 0.247 (-0.629 to 1.123) |  |
| Moderator |  | 0.14 | 0.888 | 0.002 (-0.024 to 0.028) |  |
| **No of sessions** | 7 |  |  |  | 0.070/8.315 |
| Intercept |  | 1.59 | 0.111 | 0.599 (-0.137 to 1.337) |  |
| Moderator |  | 0.89 | 0.373 | 0.009 (-0.030 to 0.011) |  |
|  |  |  |  |  |  |
